# Plasmonic phase modulator based on novel loss-overcompensated coupling between nanoresonator and waveguide

**DOI:** 10.1038/srep18660

**Published:** 2016-01-06

**Authors:** Song-Jin Im, Gum-Song Ho, Da-Jie Yang, Zhong-Hua Hao, Li Zhou, Nam-Chol Kim, Il-Gwang Kim, Qu-Quan Wang

**Affiliations:** 1 Department of Physics, Kim Il Sung University, Pyongyang, Democratic People’s Republic of Korea; 2 School of Physics and Technology, Wuhan University, Wuhan 430072, People’s Republic of China; 3 The Institute for Advanced Studies, Wuhan University, Wuhan 430072, People’s Republic of China

## Abstract

We present that surface plasmon polariton, side-coupled to a gain-assisted nanoresonator where the absorption is overcompensated, exhibits a prominent phase shift up to π maintaining the flat unity transmission across the whole broad spectra. Bandwidth of this plasmonic phase shift can be controlled by adjusting the distance between the plasmonic waveguide and the nanoresonator. For a moderate distance, within bandwidth of 100 GHz, the phase shift and transmission are constantly maintained. The plasmonic phase can be shift-keying-modulated by a pumping signal in the gain-assisted nanoresonator. A needed length in our approach is of nanoscale while already suggested types of plasmonic phase modulator are of micrometer scale in length. The energy consumption per bit, which benefits from the nano size of this device, is ideally low on the order of 10 fJ/bit. The controllable plasmonic phase shift can find applications in nanoscale Mach–Zehnder interferometers and other phase-sensitive devices as well as directly in plasmonic phase shift keying modulators.

Plasmonic modulator is a fundamental key component for merging between nanoscale electronics and ultrafast photonics and developing ultra-compact, on-chip and high-speed devices, so it has been extensively studied for last decades^1^,^2^,^3^,^4^,^5^,^6^,^7^,^8^,^9^,^10^,^11^,^12^,^13^,^14^,^15^,^16^,^17^,^18^. Surface plasmon polariton (SPP)^19^ propagating at the metal-dielectric interface has deep mode confinement, which provides possibility to reduce transverse dimension of plasmonic modulators to subwavelength scale. Metal–insulator–metal (MIM) waveguide is demonstrated to be one of the best channels to guide light with nanoscale mode confinement and low loss^20^. Recently, based on MIM waveguide, high-speed and compact plasmonic phase modulators applying Pockels electro-optical effect^9^,^11^ and compact nanomechanical plasmonic phase modulators^10^ have been experimentally demonstrated. In spite of the subwavelength transverse size and strong local field enhancement, relative modulation of refractive index of material is smaller than the unity and therefore plasmonic modulators require a longitudinal length of tens of micrometers to achieve a sufficient interaction between the traveling plasmonic and the modulating radiofrequency (RF) fields.

The small-size optical resonator with a large quality factor resonantly enhances the interaction per length at the expense of reduced optical bandwidth. The size of the resonant non-plasmonic electro-optic modulator could be reduced down to micrometer scale as the dimension of multi-wavelength traditional optical resonator^21^,^22^,^23^,^24^. A resonant silicon electro-optic modulator with a footprint of 78 μm^2^ has been experimentally demonstrated^21^. Localized surface plasmon modes in plasmonic cavity are promising for nanoscale resonator. It is expected that the longitudinal dimension could be reduced by integrating plasmonic waveguide and resonator. Plasmonic resonator coupled to the waveguide has been intensively studied theoretically and experimentally^25^,^26^,^27^,^28^,^29^,^30^,^31^,^32^,^33^,^34^,^35^,^36^,^37^,^38^,^39^,^40^,^41^ for controlling SPP such as plasmonic filters^25^,^26^,^27^, amplitude modulators and switches^3^,^15^,^28^,^29^,^30^, demultiplexers^31^,^32^,^33^,^34^, sensors^35^,^36^,^37^,^38^, slow light waveguides^39^, diodes^40^ and rectifiers^41^.

However, both propagating and localized surface plasmons suffer from material loss in the metal. Surface plasmons in plasmonic nanocavities have a small quality factor due to the loss, limiting their use as high-quality resonators. It was proposed that introduction of a gain material can compensate the loss and lead to high-quality plasmon resonances which have been implemented in applications such as surface plasmon amplification of stimulated emission of radiation (spaser)^42^,^43^,^44^, surface-enhanced Raman scattering^45^ and plasmonic switch^17^. The gain material was also introduced to plasmonic waveguides^46^ to obtain the high resonance effects such as the zero-group velocity for trapping light^47^,^48^,^49^.

In spite of the intensive and diverse works to control light in nanoscale based on the conception of the plasmonic resonator-waveguide coupling, to our knowledge, so far no work has been presented to propose and develop a nanoscale device to control plasmonic phase with deep contrast, which is important for merging between nanoscale electronics and ultrafast photonics^9^,^10^,^11^. As one can know from the temporal coupled-mode theory^50^, SPP side-coupled to a typical plasmonic resonator with internal loss experiences no phase shift on resonance. This is why the conception of the plasmonic resonator-waveguide coupling has never been proposed for high-contrast plasmonic phase modulation. Even in the case of the gain-assisted plasmonic resonator^17^, which operates between the full loss state and the loss-exactly compensated state, any transmitted SPP with significant phase shift cannot be observed. However, if the internal loss is overcompensated, the situation becomes different. Recently we studied controllable amplification and suppression of SPP side-coupled to the plasmonic resonator which operates on the loss-overcompensated state^18^.

In this paper, we propose an approach to control plasmonic phase using a gain-assisted plasmonic nanoresonator, where the internal loss is overcompensated. SPP side-coupled to the loss-overcompensated plasmonic resonator exhibits a significant phase shift up to π, maintaining the flat unity transmission spectra. Bandwidth of this plasmonic phase shift can be controlled by adjusting the distance between the plasmonic waveguide and the nanoresonator. For a moderate distance, within bandwidth of 100 GHz phase shift and transmission are constantly maintained. The plasmonic phase can be shift-keying-modulated by switching between lossy and loss-overcompensated couplings with a pumping signal in the coupled resonator. Compactness of this device is limited by the size of the plasmonic resonator which is on the order of 100 nm. The energy consumption per bit of this device is extremely low on the order of 10 fJ/bit, which meets requests for a future chip-scale optical link^51^.

## Results

### Temporal coupled-mode theory of the novel loss-overcompensated coupling

The spectral features of plasmonic waveguide-resonator systems can be investigated by the temporal coupled-mode theory^50^,


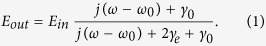


Here, *ω* and *ω*_0_ are the considered angular frequency and the resonant angular frequency, respectively. *E*_in_ and *E*_out_ are the input and output electric field strength, respectively. *γ*_0_ and *γ*_*e*_ are the decay rate and the escape rate due to the coupling between the waveguide and the resonator, respectively^50^. If we introduce a gain material into the plasmonic resonator, Eq. (1) becomes as follows,


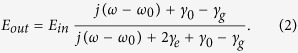


Here, *γ*_g_ is the growth rate of the field in the plasmonic resonator due to the gain of the material. The transmission coefficient can be expressed as follows,


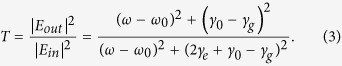


Let’s first assume that the gain is introduced to compensate the internal loss and the growth rate *γ*_g_ due to the gain is not larger than the decay rate *γ*_0_ due to the internal loss, that is *γ*_g_ < *γ*_0_. Then the phase shift of SPP due to coupling to the plasmonic resonator can be expressed as follows,


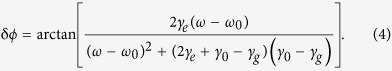


One can see δ*ϕ* = 0 on resonance from Eq. (4). This means that SPP experiences no phase shift through coupling to the plasmonic resonator.

Interestingly, in the case of loss-overcompensated state, that is *γ*_g_ > *γ*_0_, a prominent phase shift up to π is exhibited nearby the resonance. More accurately for 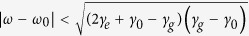, δ*ϕ* is expressed as follows,


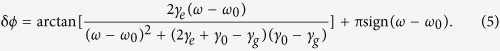


We can know from Eq. (5) that the bandwidth of the plasmonic phase shift (Δ*ω* = *ω* − *ω*_0_ at δ*ϕ* = π/2) is


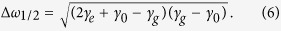


What is more interesting is in the special case of (*γ*_g_ − *γ*_0_)/*γ*_e_ = 1, where the bandwidth of the plasmonic phase shift (Eq. (6)) has the maximum value. In this special case the transmission coefficient is the unity in the whole range of wavelength *T* (*ω*) = 1 as we can know from Eq. (3). The plasmonic phase shift nearby the resonance (|*ω* − *ω*_0_| < *γ*_e_) can be expressed as follows,


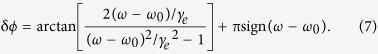


The bandwidth of the plasmonic phase shift equals to the escape rate, Δ*ω*_1/2, max_ = *γ*_e_. The escape rate *γ*_e_ is predicted to be adjusted by changing the distance *d* between the plasmonic waveguide and the coupled resonator^18^.

**Figure 1:**
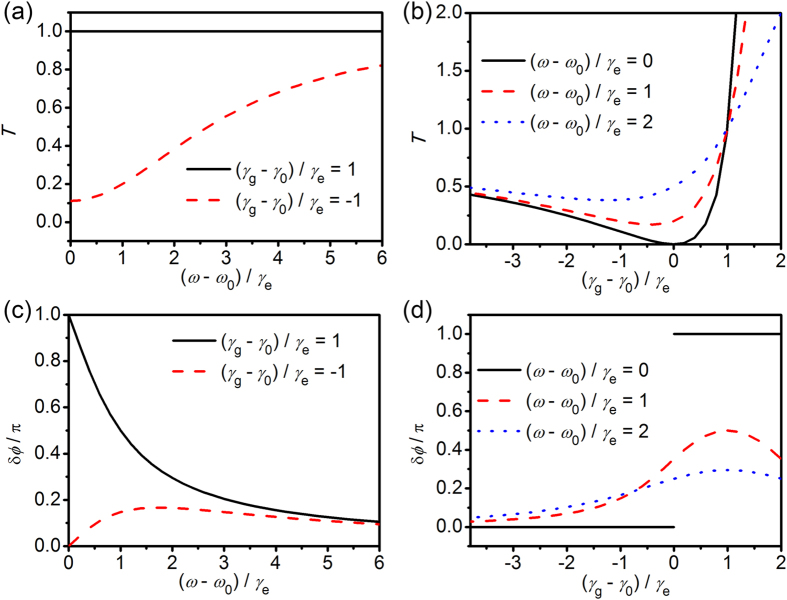
Temporal coupled-mode theory of loss-overcompensated coupling. Transmission coefficient (**a**,**b**) and phase shift (**c**,**d**) of the plasmonic waveguide-resonator system versus the normalized detuning (*ω* − *ω*_0_)/*γ*_e_ of angular frequency (**a**,**c**) and the normalized growth rate (*γ*_g_ − *γ*_0_)/*γ*_e_ (**b**,**d**), calculated by the temporal coupled-mode theory of the loss-overcompensated coupling. In (**a**,**c**), the black solid lines are for the loss-overcompensated state (*γ*_g_ − *γ*_0_)/*γ*_e_ = 1 and the red dashed lines are for the loss state (*ω* − *ω*_0_)/*γ*_e_ = −1. In (**b**,**d**), the black solid, the red dashed and the blue dotted are for the exact resonance (*ω* − *ω*_0_)/*γ*_e_ = 0, the detuning (*ω* − *ω*_0_)/*γ*_e_ = 1 and 2, respectively.

Figure 1 shows the transmission coefficient *T* and the phase shift δ*ϕ* calculated by Eq. (4) and (5). For the loss-overcompensated state, (*γ*_g_ − *γ*_0_)/*γ*_e_ = 1, the significant phase shift δ*ϕ* up to π (the black solid of Fig. 1(c)) is shown within the bandwidth *γ*_*e*_, while the transmission coefficient *T* (the black solid of Fig. 1(a)) is the unity in the whole broad spectra. However, for a loss state, (*γ*_g_ − *γ*_0_)/*γ*_e_ = −1, one can observe no phase shift (the red dashed of Fig. 1(c)) on resonance, while a small transmission (the red dashed of Fig. 1(a)) on resonance is exhibited.

One can see more clearly the sudden change (the black solid line of Fig. 1(d)) from no phase shift to π-phase shift on resonance, which is caused by a transition from a loss state to a loss-overcompensated state. The maximum phase shift is observed for the special case, (*γ*_g_ − *γ*_0_)/*γ*_e_ = 1 (the red dashed and blue dotted lines of Fig. 1(d)). One can see also that all the curves of different frequencies (the black solid, the red dashed and blue dotted lines of Fig. 1(b)) intersect at the point of (*γ*_g_ − *γ*_0_)/*γ*_e_ = 1 and *T* = 1. We would like to note that the growth rate *γ*_g_ should be smaller than (2*γ*_e_ + *γ*_0_). If *γ*_g_ is so large that *γ*_g_ > (2*γ*_e_ + *γ*_0_), the local field strength exponentially increases with time and this kind of state is not stable.

### Physical model of the nanoscale plasmonic phase modulator

**Figure 2:**
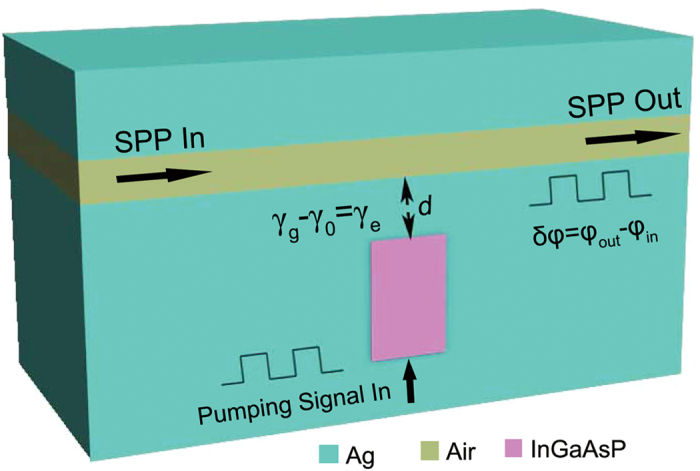
Silver-air-silver MIM waveguide side-coupled to a rectangular cavity filled with InGaAsP. The cavity has the height of 150 nm and the width of 100 nm. The thickness of the air gap of the waveguide is 50 nm. *γ*_g_ − *γ*_0_ = *γ*_e_ means the loss-overcompensated coupling.

We consider a silver-air-silver MIM waveguide side-coupled to a plasmonic rectangular resonator filled with InGaAsP, as shown in Fig. 2, where an electrical pumping is introduced to support the loss-overcompensated coupling, (*γ*_g_ − *γ*_0_)/*γ*_e_ = 1. The plasmonic rectangular resonator with the height of 150 nm and the width of 100 nm is assumed to have a resonance in the telecommunication window.

We calculate the transmission coefficient and the phase shift by numerically solving the Maxwell equation in a frequency domain. The experimental data for the permittivity spectra of silver^52^ are directly used. The permittivity of the gain medium can be expressed as *ε*_g_ = *ε*_d_ + *χ*_g_^18^, where *ε*_d_ is the permittivity of the gain medium excluding the gain transition contribution and *χ*_g_ is the gain-transition susceptibility. Here, we used *ε*_g_ = 11.38 of InGaAsP for the telecommunication wavelengths^17^.

We take account of the frequency dependency of the gain-transition susceptibility *χ*_g_. The frequency-dependent gain-transition susceptibility *χ*_g_ (*ω*) can be expressed in the two-level approximation^53^ by


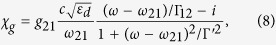


where *ω*_21_ is the gain-transition line-center, Γ_12_ is the dipole dephasing rate, *g*_21_ is the line-center gain coefficient and Γ′ is the gain-transition line width. For a sufficiently strong electric field,


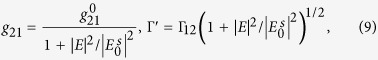


where 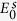 is the line-center saturation field strength and 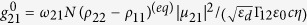 is the line-center gain coefficient for a weak electric field compared to 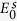. We used the dipole dephasing rate Γ_12_ = 5 × 10^13^ s^−1^ 54. It is assumed that the electric field is sufficiently weak and Γ′ = Γ_12_.

**Figure 3:**
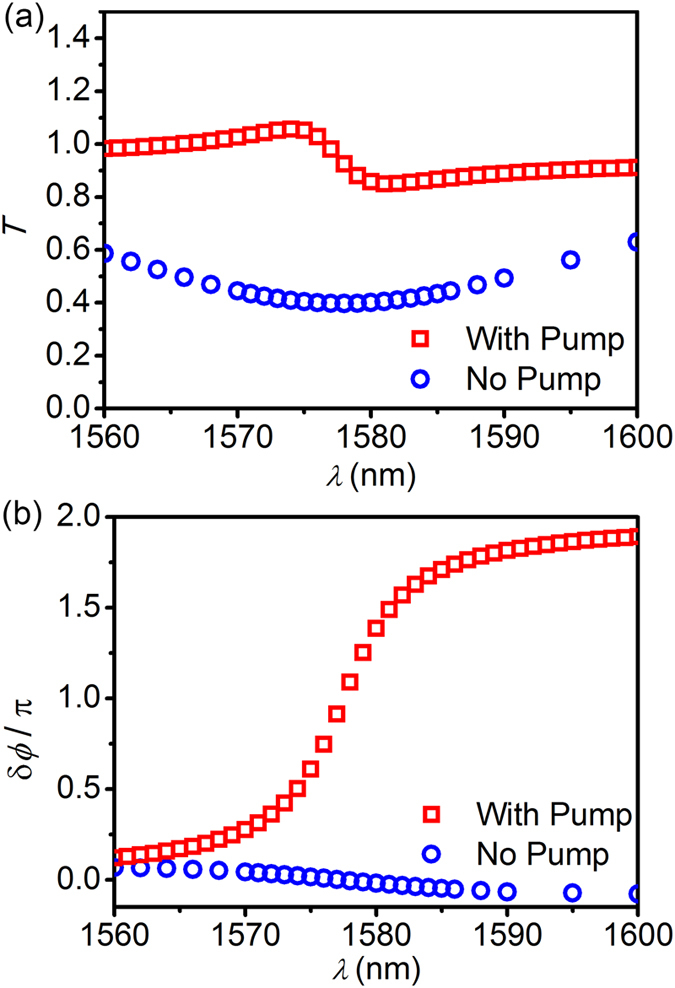
Spectra of transmission and phase shift. Transmission coefficient *T* (**a**) and phase shift δ*ϕ* (**b**) according to the wavelength in the waveguide side-coupled to the resonator in the absence of pumping and in the presence of pumping where the gain coefficient *g*_21_ = 2100 cm^−1^. The distance between the resonator and the waveguide *d* = 20 nm. Other parameters are same as in Fig. 2.

In the absence of pumping, InGaAsP exhibits an absorption and the gain coefficient is assumed to be *g*_21_ = −1230 cm^−1^ corresponding to *ε* = 11.38 + 0.1*i*^17^ by the relation 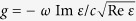
18. In this case, one can see a significant transmission (the blue circles of Fig. 3(a)), which is attributed to a very week coupling due to the strong absorption of the metal and the InGaAsP without pumping. And no significant phase shift (the blue circles of Fig. 3(b)) is observed as predicted from Eq. (4) and the red dashed line of Fig. 1(c). In the presence of pumping, InGaAsP exhibits a gain and the gain coefficient is assumed to be *g*_21_ = 2100 cm^−1^ for an appropriate pumping rate. In this case, one can see the flat transmission coefficient of the unity in the whole wavelength range (the red squares of Fig. 3(a)). This corresponds to the loss-overcompensated state, (*γ*_g_ − *γ*_0_)/*γ*_e_ = 1 (the black solid line of Fig. 1(a)). In this loss-overcompensated state, the π-phase shift on resonance (the red squares of Fig. 3(b)) is observed, as predicted from Eq. (7) and the black solid line of Fig. 1(c). This prominent phase shift of SPP maintaining its amplitude in a broad spectral range is quite promising for plasmonic phase modulation. It is noted that the realization of gain coefficients below 3000 cm^*−*1^ is feasible with current technology^17^.

**Figure 4:**
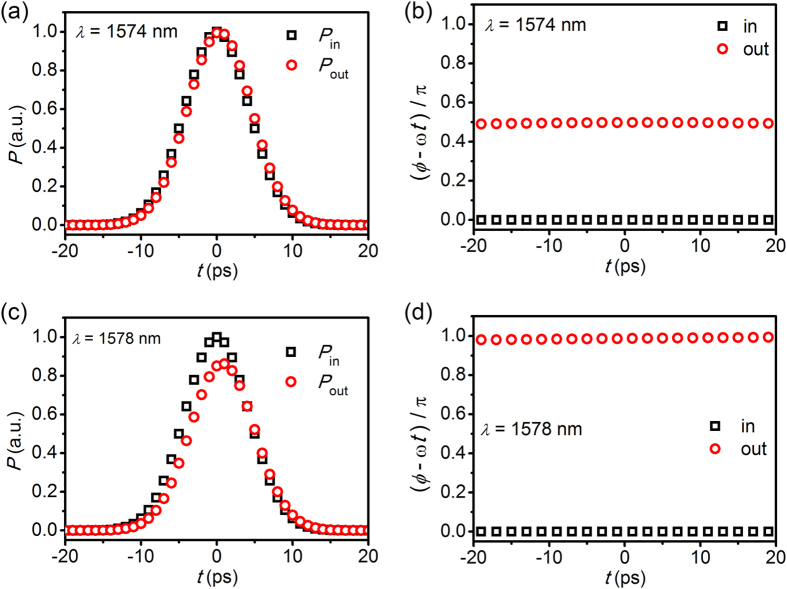
Change of pulse amplitude and phase. Temporal power (**a**,**c**) and phase (**b**,**d**) of plasmonic pulse in the waveguide side-coupled to the resonator with *d* = 20 nm. Here *g*_21_ = 2100 cm^−1^ and other parameters are same as in Fig. 2. FWHM width of the input pulse is 10 ps. The input pulses have the central wavelengths of 1574 nm (**a**,**b**) and 1578 nm (**c**,**d**). The black squares are the power and phase of the input pulse. The red circles are the power and phase of the output pulse.

We consider temporal amplitude and phase of plasmonic pulse in the waveguide side-coupled to the resonator with *d* = 20 nm. Here, *g*_21_ = 2100 cm^−1^ corresponding to the loss-overcompensated state (*γ*_g_ − *γ*_0_)/*γ*_e_ = 1. The full-width at half maximum (FWHM) of the pulse is 10 ps and the central wavelength is 1574 nm (Fig. 4(a,b)) and 1578 nm (Fig. 4(c,d)). The wavelength of 1578 nm corresponds to the exact resonance. In both cases, the plasmonic pulses almost keep their amplitude and pulse shape (Fig. 4(a),(c)). Only the phase of the pulse is shifted by π/2 (Fig. 4(b)) and by π (Fig. 4(d)), respectively.

**Figure 5:**
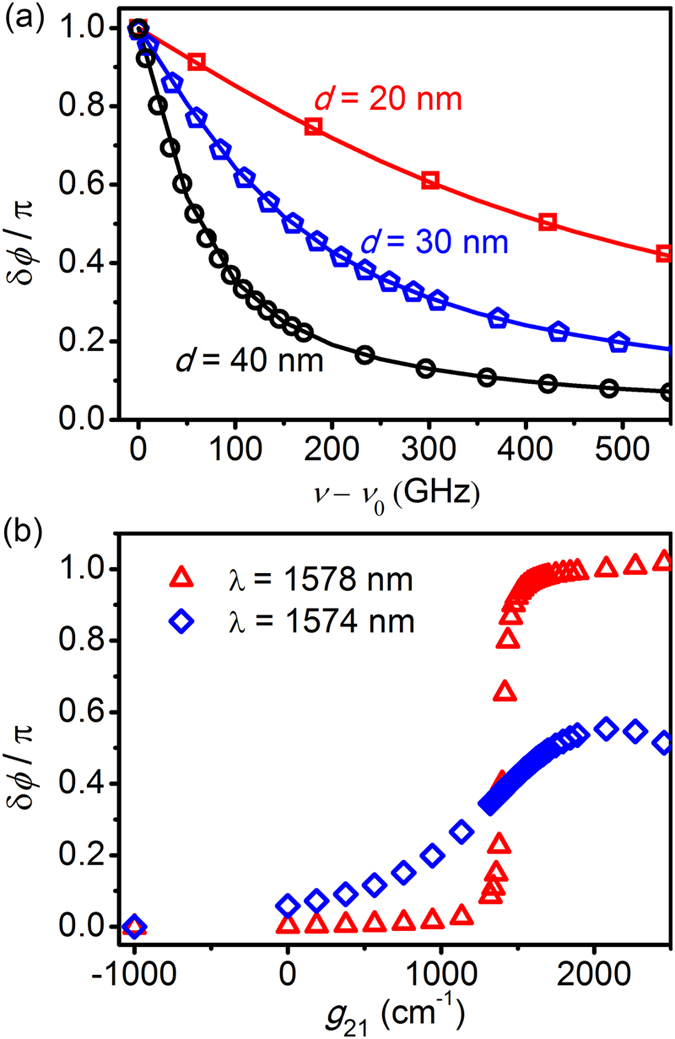
Control of plasmonic phase shift. (**a**) Plasmonic phase shift δ*ϕ* according to the detuning of the frequency *ν* from the resonance frequency *ν*_0_ in the waveguide side-coupled to the resonator with different distances *d* = 20 nm (red squares), *d* = 30 nm (blue pentagons) and *d* = 40 nm (black circles). The red, blue and black solid lines are the results calculated by Eq. (7). The gain coefficients are selected appropriately to satisfy the condition (*γ*_g_ − *γ*_0_)/*γ*_e_ = 1. Other parameters are same as in Fig. 2. (**b**) Plasmonic phase shift according to the gain coefficient *g*_21_ in the waveguide side-coupled to the resonator with the distance *d* = 20 nm.

In the previous section, we predicted that the phase shift spectrum has the maximum bandwidth of *γ*_e_ for the case of (*γ*_g_ − *γ*_0_)/*γ*_e_ = 1. The bandwidth *γ*_e_ is controllable by changing the distance *d* between the resonator and the waveguide. Simulation results of phase shift spectrum for the loss-overcompensating coupling (*γ*_g_ − *γ*_0_)/*γ*_e_ = 1 with different *d* (Fig. 5(a)) confirms that the bandwidth of the phase shift becomes broader for the smaller *d* which leads to a strong coupling and a large escape rate *γ*_e_. It also shows that the simulation results (the scattered shapes of Fig. 5(a)) perfectly coincide with the results calculated by Eq. (7) (the solid lines of Fig. 5(a)) based on the temporal coupled mode theory. A bandwidth of the phase shift more than 100 GHz is achievable for a moderate distance *d*.

The broad band plasmonic phase shift can be controlled by changing the parameter (*γ*_g_ − *γ*_0_)/*γ*_e_ as predicted in the previous section. Here, we consider the plasmonic phase shift in the waveguide side-coupled to the resonator according to the gain coefficient of InGaAsP. The on-resonance phase shift (the red triangles of Fig. 5(b)) varies from the zero to π for different gain coefficients. This transition between in-phase and π-phase shift can be applied to binary phase shift keying (BPSK) modulators. If the considered central wavelength is appropriately detuned from the exact resonance, phase shift can be controlled from the zero to π/2 (the blue diamonds of Fig. 5(b)), which is needed for quadrature phase shift keying (QPSK) modulators^9^,^24^.

It is noted that through all the calculation the frequency-dependency of the gain coefficient is taken into account, but here it does not significantly influence the phase shift and the transmission because of the relatively high dipole dephasing rate on the order of 10 THz^54^ compared to *γ*_e_ on the order of 100 GHz.

## Discussions and Conclusions

We argue that this conception of plasmonic phase modulator based on the novel loss-overcompensated coupling is the first proposed nanoscale optical phase modulator with high contrast and could be a good candidate for the on-chip broadband low-power consumption optical interconnects. The Pockels effect-based plasmonic phase modulators^9^,^11^ require the length of 10 μm because a multi-wavelength propagation is needed to carry out a significant phase shift with a sub-unity index modulation in spite of the enhanced nonlinear interaction. The nanoelectromechanical effect-based one^10^ reduces the length, but still has the length on the order of μm. The sub-wavelength-scale plasmonic amplitude switches and modulators^3^,^15^,^17^,^28^,^29^,^30^ based on the typical plasmonic resonator-waveguide coupling were proposed. However, to our knowledge, a high-contrast phase modulator using the plasmonic resonator-waveguide coupling has never been proposed despite both exciting topics of the plasmonic phase modulator and the plasmonic resonator-waveguide coupling have been intensively studied as mentioned in the introduction. This is because no phase shift on resonance is observed for the typical lossy resonator-waveguide coupling. The significant phase shift of transmitted SPP on resonance is uniquely observed for the novel loss-overcompensated coupling. The loss-overcompensated coupling between the plasmonic resonator and waveguide also exhibits the flat unity transmission across the whole spectral range^18^, which is promising for a phase modulator. Efficient integration of this kind of MIM structure on Si chip has been discussed^3^ and experimentally demonstrated^55^. The bandwidth of phase shift for this approach is more than 100 GHz for a moderate distance between the waveguide and the nanoresonator. The modulation speed is limited to the order of 5 GHz by the carrier life time which is on the order of 0.2 ns^17^. This modulation speed is lower than that of the Pockels effect-based device^9^,^11^, but far higher than that of the nanoelectromechanical effect-based device^10^. Moreover, the modulation speed per area is very high because the length of this modulator is on the order of 100 nm, while other suggested plasmonic phase modulators have lengths on the order of 10 μm. The low power consumption on the order of 50 μW^17^ results in ideally low energy consumption per bit on the order of 10 fJ/bit which is promising for future optical interconnects to chips^51^.

We have proposed a new conception of nanoscale plasmonic phase modulator. This plasmonic phase modulator is based on the novel loss-overcompensated coupling between the plasmonic resonator and waveguide. The needed length is on the order of 100 nm. Bandwidth of the phase shift reaches more than 100 GHz. The modulation speed is limited only by the carrier life time. The low power consumption on the order of 50 μW and the low energy consumption per bit on the order of 10 fJ/bit is predicted. We expect that the speed limitation by the carrier life time can be avoided if one uses an ultrafast nonlinear absorption maintaining a sufficient gain. This nanoscale plasmonic phase modulator can find applications in nanoscale Mach–Zehnder interferometers and other phase-sensitive devices as well as directly in plasmonic phase shift keying modulators. All the realistic parameters are used here and the realization of this approach appears feasible with current material and lithography technology.

## Methods

The temporal coupled-mode theory^50^ is used to predict the properties of the loss-overcompensating coupling between the resonator and the waveguide.

We calculated the transmission coefficient and the phase shift in the realistic physical model by numerically solving the Maxwell equation in a frequency domain^17^,^18^. The temporal response to the input pulse is calculated by numerically superpositioning responses to Fourier components of the temporal pulse.

Here, we used the experimental data for the permittivity spectra of silver^52^ and the real part of the permittivity of InGaAsP, Re*ε*_InGaAsP_ = 11.38^17^ in the telecommunication window. The frequency-dependence of the gain-transition susceptibility *χ*_*g*_(*ω*) was taken into account in the two-level approximation^18^,^53^.

## Additional Information

**How to cite this article**: Im, S.-J. *et al.* Plasmonic phase modulator based on novel loss-overcompensated coupling between nanoresonator and waveguide. *Sci. Rep.* **6**, 18660; doi: 10.1038/srep18660 (2016).
